# DRG2 as a Biomarker to Enhance the Predictive Efficacy of PD-L1 Immunohistochemistry Assays

**DOI:** 10.3390/biomedicines13010056

**Published:** 2024-12-29

**Authors:** Muralidharan Mani, Seong Hee Choi, Hyuk Nam Kwon, Jeong Woo Park

**Affiliations:** 1 Department of Biochemistry, University of Wisconsin-Madison, Madison, WI 53715-1218, USA; mmani3@wisc.edu; 2 RopheLBio, B102, Seoul Forest M Tower, Seoul 04778, Republic of Korea; nacchal2@naver.com; 3 Department of Biological Sciences, University of Ulsan, Ulsan 44610, Republic of Korea; hnkwon@ulsan.ac.kr; 4 Basic-Clinic Convergence Research Institute, University of Ulsan, Ulsan 44610, Republic of Korea

**Keywords:** DRG2, PD-L1, endosomal trafficking, ICI, predictive efficacy

## Abstract

PD-L1 immunohistochemistry (IHC) assays are used as a companion diagnostic for immunotherapy with immune checkpoint inhibitors (ICIs). However, despite the association between PD-L1 expression and clinical benefit from ICIs, the PD-L1 IHC assay is not sufficiently accurate in predicting response to ICIs; some patients with high PD-L1 expression do not respond to ICIs. Recently, researchers provided insights into why some patients with high PD-L1 expression fail to respond to ICIs. They discovered that DRG2 is a critical regulator of PD-L1 endosomal trafficking in cancer cells, which is essential for the proper localization of PD-L1 on the cell surface. Although DRG2-depleted cells express high levels of PD-L1 and are PD-L1 IHC-positive, the PD-L1 sequestered in early endosomes does not respond to ICIs. Therefore, a companion diagnostic combining DRG2 expression with a PD-L1 IHC assay may improve the therapeutic response to PD-1/PD-L1 ICIs.

Programmed Death-Ligand 1 (PD-L1) is an intriguing surface protein found on tumor cells. Its presence plays a crucial role in how these cells interact with the immune system, influencing the body’s ability to recognize and combat cancer. Understanding PD-L1 is essential in the fight against cancer, as it paves the way for innovative treatments and therapies. Recently, Choi et al. [1] reported that developmentally regulated GTP-binding protein 2 (DRG2) is required for PD-L1 localization on the cancer cell surface. On the cancer cell surface, it interacts with the PD-1 receptor on T-cells, inhibiting anti-tumor immunity by triggering immune checkpoint responses [2]. Monoclonal antibodies that block PD-1/PD-L1 interactions, known as immune checkpoint inhibitors (ICIs), reinvigorate the cancer-killing activity of T-cells, leading to improved tumor immune surveillance. This innovative immunotherapy demonstrates remarkably durable and sustained responses across various tumor types, with some patients remaining free from cancer progression for many years [3,4]. However, despite remarkable success in a subset of patients, currently approved ICIs have shown objective responses in only about 20–30% of treated tumors [5], emphasizing the crucial need to identify biomarkers to select appropriate patients for ICI treatment.

Numerous studies across various cancer types have demonstrated a significant positive correlation between the expression levels of PD-L1 on tumor cells and the therapeutic response to ICIs. This relationship suggests that higher PD-L1 expression may enhance ICI effectiveness, positioning PD-L1 as a crucial biomarker for predicting patient outcomes and guiding treatment strategies in cancer immunotherapy [6,7,8,9]. Based on these findings, PD-L1 expression on tumor or immune cells has emerged as a predictive biomarker for sensitivity to ICIs, and immunohistochemistry (IHC) assays to evaluate PD-L1 expression have been approved by the US FDA as a companion diagnostic for immunotherapy with ICIs in specific tumor types [10,11]. However, despite the association between PD-L1 expression and clinical benefit from ICIs in various cancer types, the PD-L1 IHC assay is not sufficiently accurate or reliable in predicting response to ICIs [12,13,14]. Some patients with high PD-L1 expression do not respond to ICIs [6], highlighting potential limitations in predicting therapeutic response through PD-L1 assessment [12,15].

Recently, Choi et al. [1] identified the role of DRG2 in the endosomal trafficking of PD-L1, providing insights into why some patients with high PD-L1 expression do not respond to ICIs (Figure 1). PD-L1 on the cell surface is continuously internalized via endocytosis and directed to Rab5-positive early endosomes. From there, PD-L1 is either sorted toward Rab7-positive lysosomes for degradation or Rab11-positive recycling endosomes for recycling back to the cell surface [16,17,18,19]. DRG2 controls the endosomal trafficking of PD-L1, which is directly linked to its regulation of Rab5 activity. Rab5, localized to early endosome, is crucial for early endocytosis. However, for endosomal trafficking to lysosomes or recycling endosomes, Rab5 must be deactivated on early endosomes [20,21]. Previously, Mani et al. [22] reported that DRG2 plays an essential role in deactivating Rab5 by facilitating the interaction between Rab5 and RabGAP5. DRG2 depletion impairs Rab5 deactivation on early endosomes, blocking trafficking to recycling endosomes or lysosomes, and causing surface proteins to accumulate in early endosomes [22,23]. Consistently, Choi et al. [1] demonstrated that while DRG2 depletion enhanced PD-L1 expression, it induced the accumulation of PD-L1 in Rab5-positive early endosomes.

Given that conventional IHC assay lacks accuracy due to cytoplasmic PD-L1 staining interfering with the assessment of cell membrane PD-L1, DRG2-depleted tumors with endosomal accumulation of PD-L1 may still test positive by IHC. However, since ICIs blocking PD-1/PD-L1 interaction are effective only on cell surface PD-L1 [16,24,25], the endosomal accumulation of PD-L1 in DRG2-depleted cells may limit the efficacy of anti-PD-1 therapies. Consistently, Choi et al. [1] provided evidence supporting this hypothesis: PD-L1 in DRG2-depleted cancer cells showed reduced binding to recombinant PD-1 and exhibited defects in inhibiting T-cell activity. Additionally, mice bearing DRG2-depleted melanoma did not respond to anti-PD-1 treatment, and data analysis of a melanoma patient cohort undergoing anti-PD-1 treatment [26] revealed that low DRG2 protein expression correlated with poor clinical response in melanoma patients. However, DRG2 RNA levels did not correlate with clinical response in a cohort of melanoma patients treated with anti-PD-L1 therapy [27]. DRG2 transcription is upregulated by the transcription factor stimulating protein 1 (Sp1) [28], and DRG2 protein stability is enhanced through interaction with RWDD1 [29,30]. However, it remains unclear whether these mechanisms are the primary regulators of DRG2 in cancer cells.

Several factors, in addition to DRG2, regulate the recycling and lysosomal degradation of PD-L1. For instance, CMTM6 interacts with PD-L1 at the plasma membrane, facilitating the recycling of endocytosed PD-L1 [16]. TRAPPC4 interacts with PD-L1 in recycling endosomes and, like CMTM6, promotes PD-L1 recycling, diverting it away from the lysosomal degradation [31]. Depletion of either CMTM6 or TRAPPC4 reroutes endocytosed PD-L1 toward lysosomal degradation of PD-L1, leading to a decrease in surface PD-L1 level in cancer cells [16,27]. HIP1R, on the other hand, prevents the recycling of endocytosed PD-L1 and instead directs it to the lysosome for degradation [32]. Depletion of HIP1R enhances the recycling of endocytosed PD-L1, resulting in its accumulation at the cell surface [31]. While these molecules regulate the endosomal trafficking of PD-L1, they mainly influence the balance between recycling back to the cell surface and lysosomal degradation. Therefore, unlike DRG2, they do not cause the accumulation of PD-L1 in endosomes.

In conclusion, Choi et al. [1] identified DRG2 as a critical regulator of PD-L1 endosomal trafficking in cancer cells, which is essential for the proper localization of PD-L1 on the cell surface and, consequently, for an effective response to ICIs. DRG2-depleted cancer cells exhibit defects in the trafficking of PD-L1 from early endosomes to recycling endosomes and lysosomes, resulting in PD-L1 accumulation in early endosomes rather than on the cell surface. As a result, despite expressing high levels of PD-L1 and being PD-L1 IHC-positive, these cells are unable to effectively interact with ICIs due to the sequestration of PD-L1 within early endosomes. Conversely, cancer cells with high DRG2 expression display proper localization of PD-L1 on the cell surface, thereby facilitating a more effective response to ICIs. Cell surface PD-L1 can be detected using flow cytometric analysis of non-permeabilized cells. However, this method is limited to liquid tumors and fresh tissue samples from solid tumors, making it unsuitable for paraffin-embedded solid tumor samples. Therefore, the selection of cancer patients with high levels of both DRG2 and PD-L1 proteins through a companion diagnostic approach that combines DRG2 expression assessment with PD-L1 IHC assay may improve the therapeutic outcomes with PD-1/PD-L1 ICIs. Similar to PD-L1, the quantitative evaluation of DRG2 expression using IHC and determination of a DRG2 cutoff value can be challenging. However, we believe this issue can be addressed by developing an artificial intelligence (AI)-based method for quantifying DRG2 IHC expression and establishing DRG2 scoring cutoffs. It is worth noting that Choi et al. [1] determined the role of DRG2 in PD-L1 endosomal trafficking specifically in melanoma cells. It remains unclear whether DRG2 plays a similar role in other cancer types. However, previous studies have reported that DRG2 regulates the endosomal trafficking of surface receptors, such as the transferrin receptor and epidermal growth factor receptor, in breast cancer and cervical cancer cells [22,23]. These findings suggest that DRG2′s role in PD-L1 trafficking may be generalizable to other cancer types. DRG2 is a GTP-binding protein that alternates between an active GTP-bound form and an inactive GDP-bound form. However, it is currently unknown which form is responsible for regulating PD-L1 endosomal trafficking, and no method exists to distinguish between these two forms. Additionally, there is no information on DRG2 mutations that may disrupt its role in PD-L1 trafficking. Thus, merely quantifying DRG2 expression may not be sufficient for use as a companion diagnostic. Further studies are needed to identify which DRG2 form regulates PD-L1 endosomal trafficking and to evaluate the correlation between DRG2 expression and the response to PD-1/PD-L1 ICIs in PD-L1 IHC-positive patients across various cancer types, including melanoma, breast cancer, cervical cancer, and others.

## Figures and Tables

**Figure 1:**
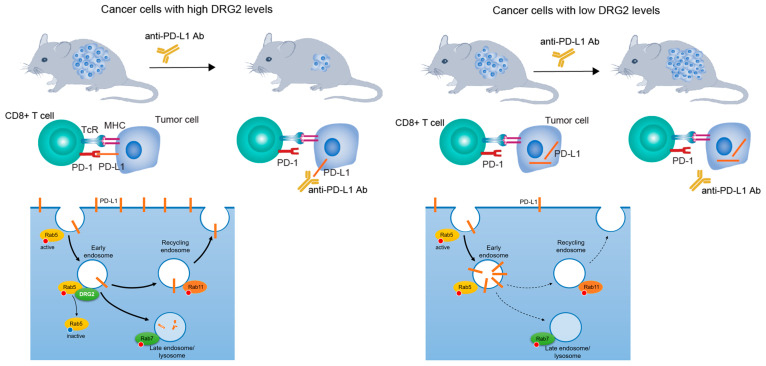
DRG2 is essential for cell surface localization of PD-L1 and the response to ICIs. PD-L1 IHC assays have been approved as a companion diagnostic for immunotherapy with ICIs. However, PD-L1 IHC assays are not sufficiently accurate or reliable in predicting response to ICIs, and some patients with high PD-L1 expression do not respond to these therapies. Choi et al. [1] provided insights into why some patients with high PD-L1 expression do not respond to ICIs. DRG2 is required for the endosomal trafficking of PD-L1 from early endosomes to recycling endosomes or lysosomes [22,23]. DRG2-depleted cancer cells show defects in trafficking of PD-L1 from early endosomes to recycling endosomes and lysosomes, leading to PD-L1 accumulation in early endosomes. Although DRG2-depleted cells express high levels of PD-L1 and are PD-L1 IHC positive, the PD-L1 accumulated in early endosomes does not respond to ICIs [1]. Therefore, a companion diagnostic combining DRG2 expression with PD-L1 IHC assays may enhance the therapeutic response to PD-1/PD-L1 ICIs. Thick solid arrows at the bottom represent the trafficking of endosomes, while the thin dashed arrows indicate the inhibition of endosomal trafficking.
